# Inspiratory muscle training as adjuvant therapy in obstructive sleep apnea: a randomized controlled trial

**DOI:** 10.1590/1414-431X2022e12331

**Published:** 2022-10-03

**Authors:** L.M. de Azeredo, L.C. de Souza, B.L.S. Guimarães, F.P. Puga, N.S.C.S. Behrens, J.R. Lugon

**Affiliations:** 1 Programa de Pós-Graduação em Ciências Médicas, Faculdade de Medicina, Universidade Federal Fluminense, Niterói, RJ, Brasil; 2 Faculdade de Fisioterapia, Universidade Estácio de Sá, Niterói, RJ, Brasil; 3 Hospital Naval Marcílio Dias, Rio de Janeiro, RJ, Brasil; 4 Divisão de Nefrologia, Departamento de Medicina, Faculdade de Medicina, Universidade Federal Fluminense, Niterói, RJ, Brasil

**Keywords:** Sleep, Obstructive sleep apnea, Respiratory muscle training, Breathing exercise

## Abstract

The aim of this randomized controlled trial was to analyze the effects of an inspiratory muscle training (IMT) program on apnea and hypopnea index (AHI), inspiratory muscle strength, sleep quality, and daytime sleepiness in individuals with obstructive sleep apnea (OSA), whether or not they used continuous positive airway pressure (CPAP (+/−) therapy. The intervention group underwent IMT with a progressive resistive load of 40-70% of the maximum inspiratory pressure (PImax) for 30 breaths once a day for 12 weeks. The control group was submitted to a similar protocol, but with at a minimum load of 10 cmH_2_O. Changes in the AHI were the primary outcome. PImax was measured with a digital vacuometer, daytime somnolence was measured by the Epworth sleepiness scale (ESS), and the quality of sleep by the Pittsburgh Sleep Quality Index (PSQI). CPAP use was treated as a confounder and controlled by stratification resulting in 4 subgroups: IMT−/CPAP−, IMT−/CPAP+, IMT+/CPAP−, and IMT+/CPAP+. Sixty-five individuals were included in the final analysis. Significant variations were found in the 4 parameters measured throughout the study after the intervention in both CPAP− and CPAP+ participants: PImax was increased and AHI was reduced, whereas improvements were seen in both ESS and PSQI. The twelve-week IMT program increased inspiratory muscle strength, substantially reduced AHI, and had a positive impact on sleep quality and daytime sleepiness, whether or not participants were using CPAP. Our findings reinforce the role of an IMT program as an adjunct resource in OSA treatment.

## Introduction

Obstructive sleep apnea (OSA) has been associated with clinical impairments, including excessive daytime sleepiness, cardiovascular, metabolic, and cognitive problems, increased risk of occupational and automobile accidents, and reduced quality of sleep and life expectancy (1,2). In the last decades, there has been an increase in this condition (3,4), associated with obesity, aging, craniofacial anatomy, and physical inactivity (5–57).

The gold standard for treating OSA is the use of continuous positive airway pressure (CPAP) to reduce clinical complications, but its adherence may be low in mild cases (8,9). Supportive treatments have been proposed to reduce the apnea and hypopnea index (AHI), which includes aerobic activities, oropharyngeal exercises, and recently, specific inspiratory muscle training (IMT) with a resistive spring load device (10).

A systematic review by Mendelson et al. (11) has suggested that physical exercise can improve sleep efficiency and reduce daytime sleepiness and the severity of OSA by 28%, even in the absence of the body mass index (BMI) reduction. These findings can be explained by increased strength and endurance of the upper airways dilator muscles, and reduced fluid transfer from the lower limbs to the rostral region during recumbent periods, resulting in an increased cross-sectional area of the upper airway lumen, decreased nasal resistance, and increased respiratory stability during deep sleep (11–15).

The use of muscle training with resistance loads specifically directed at the inspiratory muscles can be accompanied by an improvement in the performance of the main and accessory respiratory muscles and a reduction in the tendency to upper airway collapse during sleep (16,17). These effects may allow a reduction in AHI and an improvement in sleep quality, resulting in positive effects on daytime sleepiness, without changing BMI and neck circumference (18–22).

A limited number of studies have reported the use of IMT in patients with OSA, and the subject remains a matter of controversy (18–23). For example, some studies reported reduction in AHI (19,20,22), whereas others only found benefits regarding sleep quality (18).

The present study aimed to analyze the effects of a 12-week IMT program on AHI values and OSA severity. Changes in inspiratory muscle strength, daytime sleepiness, and sleep quality were also monitored.

## Material and Methods

### Design and enrollment

A randomized controlled clinical trial (RCT) was performed in individuals with OSA diagnosed by polysomnography. Subjects were selected from the otorhinolaryngology clinics at the Marcílio Dias Naval Hospital (Rio de Janeiro) and from the private medical offices. Randomization was simple, with an allocation ratio of 1:1, using computer-generated random numbers, following recommendations from the Consolidated Standards of Reporting Trials (24). Blinding was feasible for the polysomnography technician, the participants, and the researcher in charge of data analysis.

Inclusion criteria were age >18 years and the diagnosis of OSA with AHI >5/h on polysomnography. The exclusion criteria comprised: acute or chronic lung disease, systolic blood pressure >140 mmHg or diastolic blood pressure >90 mmHg, heart failure, kidney failure, neurological or psychiatric diseases, use of drugs that could influence muscle performance, illnesses that could hinder adherence to the research protocol, speech therapy, or respiratory physiotherapy in the last three months, orthodontic therapy with an intraoral device, and body mass index (BMI) ≥40 kg/m^2^. CPAP use was not enlisted as an exclusion criterion and patients on CPAP before enrollment were maintained in such therapy.

The project was approved by the Naval Hospital's Ethics Committee under the Approval Number 1.315.924 and registered in ClinicalTrials.gov under Number 04457583. An informed consent was obtained from every patient.

### Procedures

First, all participants had their PImax measured by a digital vacuometer (MVD 300 model, Globalmed, Brazil) according to a previous protocol (25). The following forms were completed with the support of the research team: the Epworth daytime Sleepiness Scale, ESS (26) and the Pittsburgh Sleep Quality Index, PSQI (27). The participants also underwent a polysomnography (EMSA System, Brain Net BNT-EEG model, LYNX/EMSA, Brazil) by a polysomnography technician blinded for the participant's group. The intervention group performed an IMT program (one session per day for 12 weeks) with a portable device with a linear pressure resistor and designed for individual use (POWERbreathe^®^ classic, UK) (28,29). The daily IMT sessions consisted of 30 breaths. The initial load was set to ∼40% of PImax. After two weeks, the resistance was increased by 10 cmH_2_O weekly until the 5th week, totaling three adjustments. From then on, the resistance level was kept constant. The control group underwent a similar IMT program but maintained the minimum load (10 cmH_2_O) for the entire training period. Participants were not informed whether they belonged to the intervention or control group. Irrespective of the group they belong to, participants with an AHI >15/h received an indication for gold-standard treatment with nocturnal CPAP, but none of the participants initiated CPAP therapy during the study.

At the end of every four weeks, participants attended the physical therapy service at the Naval Hospital for follow-up and reevaluation. Polysomnography was repeated at the end of the study.

All participants were instructed on the objectives and methods of the study. To improve the adherence of subjects to IMT, subjects received detailed written instructions of the protocol. At each monthly re-evaluation visit, they were asked to perform the training as they did at home. The same physiotherapist was in charge of the control and intervention groups.

### Outcomes

Change in AHI was the primary outcome reported as absolute number and as percent reduction of severe cases of OSA. Secondary outcomes included changes in inspiratory muscle strength, daytime sleepiness, and sleep quality.

### Statistical analysis

The study analyzed a convenience sample of patients recruited from April 2017 to May 2020. A researcher blinded to group identity performed the data analysis. The distribution pattern of continuous variables was assessed by the Kolmogorov-Smirnov test. Data with Gaussian distribution are reported as means±SD; otherwise, median and interquartile range were used. Categorical variables are reported as frequencies. Differences between baseline and final values were assessed by the signed rank (Wilcoxon) test and between groups, by the Kruskall-Wallis ANOVA complemented by the Conover test. Differences between frequencies were tested using the chi-squared or the Fisher test as appropriate. The use of CPAP was treated as a confounder and controlled for using stratification. Values of P<0.05 were considered significant. The statistical analysis was performed using the SPSS statistics program, version 18.0 (IBM, USA).

## Results

**Figure 1:**
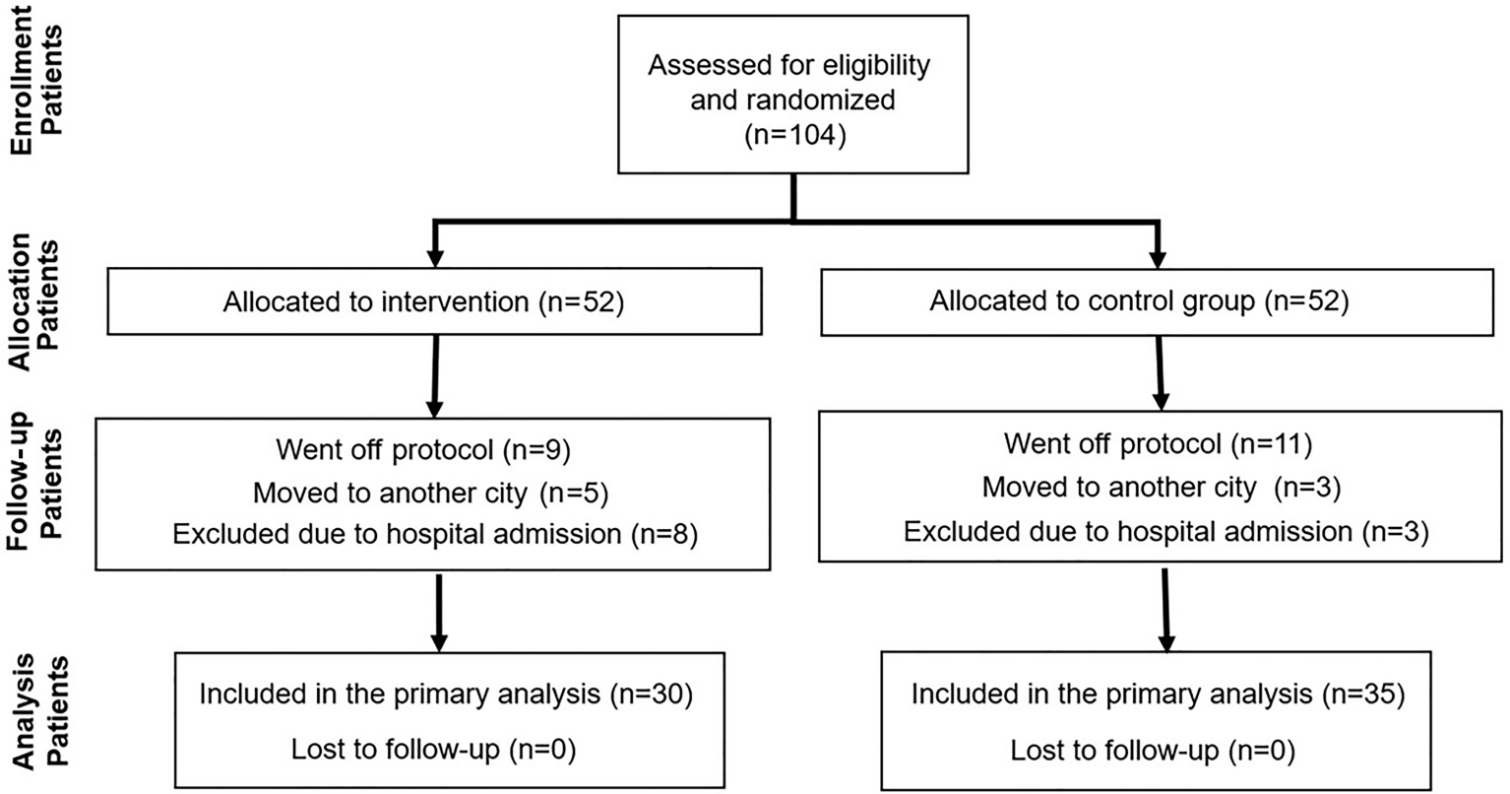
Flow diagram of participants in the study.

A flow diagram of the subjects' enrollment in the study is depicted in Figure 1. Eight patients in the intervention group and three in the control group were hospitalized due to reasons not related to the muscle training program. Thirty-five participants in the control group and 30 in the intervention group were included in the final analysis. The general characteristics of the 65 participants are shown in Table 1. At the time of study entry, 22 of the patients were already on CPAP therapy, which was not terminated. None of the participants started CPAP during the study period. Data are presented stratified by use of CPAP at entrance.

**Table 1:** General characteristics of baseline participants.

Variables	Control		Inspiratory muscle training	
	CPAP−	CPAP+	CPAP−	CPAP+
n	21	14	22	8
Age, years	55±15	55±15	63±15	67±13
Male, n (%)	14 (67)	7 (50)	13 (59)	4 (50)
Skin color (W/NW), n (%)	17/4 (81/19)	11/3 (79/21)	19/3 (86/14)	8/0 (100/0)

Data for age are reported as means±SD. CPAP(+/-): with or without continuous positive airway pressure; W: white; NW: non-white.

At maximal load, the mean increase in applied load was 66% in the intervention group and 0% in the control group. Data regarding values of BMI, neck circumference, PImax, AHI, ESS, and PSQI at baseline and study closing in the 4 subgroups are shown in Table 2. At baseline, statistically significant differences between subgroups were restricted to the AHI. Baseline values for CPAP users were statistically higher than for non-CPAP users in both groups: 44 (15-77) *vs* 20 (14-28) events/h and 53 (31-63) *vs* 29 (21-34) events/h for the control and IMT groups, respectively. Also, baseline AHI values in CPAP users of the IMT group were higher than those of the non-CPAP users of the control group, 53 (31-63) *vs* 20 (14-28) events/h.

**Table 2:** Comparison of the scores at baseline and the end of the inspiratory muscle training (IMT) program in the studied groups.

	Control				Inspiratory muscle training			
	CPAP− (n=21)		CPAP+ (n=14)		CPAP− (n=22)		CPAP+ (n=8)	
	Baseline	Follow-up	Baseline	Follow-up	Baseline	Follow-up	Baseline	Follow-up
PImax, cmH_2_O	109(82-132)	110(85-132)	95(77-109)	100(80-125)	93(66-120)	129(79-148)*	82(62-104)	95(77-121)*
AHI, events/h	20(14-28)	16(13-26)	44(15-77)^φ^	38(13-52)	29(21-34)	21(17-32)*	53(31-63)^δφ^	17(15-40)*
Epworth Scale	11(9-15)	9(7-12)	9(7-15)	6(4-8)*	8(5-11)	4(4-10)*	14(7-17)	6(5-9)*
PSQI	7(5-10)	7(5-9)	7(5-9)	6(4-7)	6(4-9)	5(3-7)*	9(7-11)	4(4-6)*
Neck circumference, cm	40.2±3.6	39.9±4.4	40.0±5.7	39.8±5.1	38.3±4.8	40.3±11.1	40 .1±5.4	40.6±5.6
Body mass index, kg/m^2^	31.2 ±5.2	32.1±7.7	31.8±6.7	32.1±6.6	29.8±5.2	29.5±5.1	33.3±4.5	33.0±3.9

Data are reported as medians and interquartile range or mean±SD. ^φ^P<0.05 *vs* baseline CPAP− in the same group; ^δ^P<0.05 *vs* baseline CPAP− in the control group; *P<0.05 *vs* baseline of the same subgroup (signed rank (Wilcoxon) test, Kruskall-Wallis ANOVA complemented by the Conover test, and chi-squared or Fisher test). CPAP(+/-): with or without continuous positive airway pressure; Pimax: maximum inspiratory pressure; AHI: apnea-hypopnea index; PSQI: Pittsburg Sleep Quality Index.

A statistically significant increase in PImax at the end of the study was only observed in the subgroups of the intervention group: 93 (66-120) *vs* 129 (79-148) cmH_2_O (P<0.001) and 82 (62-104) *vs* 95 (77-121) cmH_2_O (P= 0.017) for non-CPAP users and CPAP users, respectively. The median AHI values at study closing were significantly reduced in the intervention subgroups: 29 (21-34) *vs* 21 (17-32) events/hour (P=0.015) and 53 (31-63) *vs* 17 (15-40) events/hour (P=0.017) for non-CPAP users and CPAP users, respectively. A statistically significant reduction in the ESS was identified in CPAP users in the control group, 9 (7-15) *vs* 6 (4-8) (P=0.003), and both subgroups of the intervention group, 8 (5-11) *vs* 4 (4-10) (P=0.022), and 14 (7-17) *vs* 6 (5-9) (P=0.027) for non-CPAP users and CPAP users, respectively.

A significant improvement in sleep quality was only seen in the intervention group: 6 (4-9) *vs* 5 (3-7) (P=0.014) and 9 (7-11) *vs* 4 (4-6) (P=0.018) for non-CPAP users and CPAP users, respectively.

**Figure 2:**
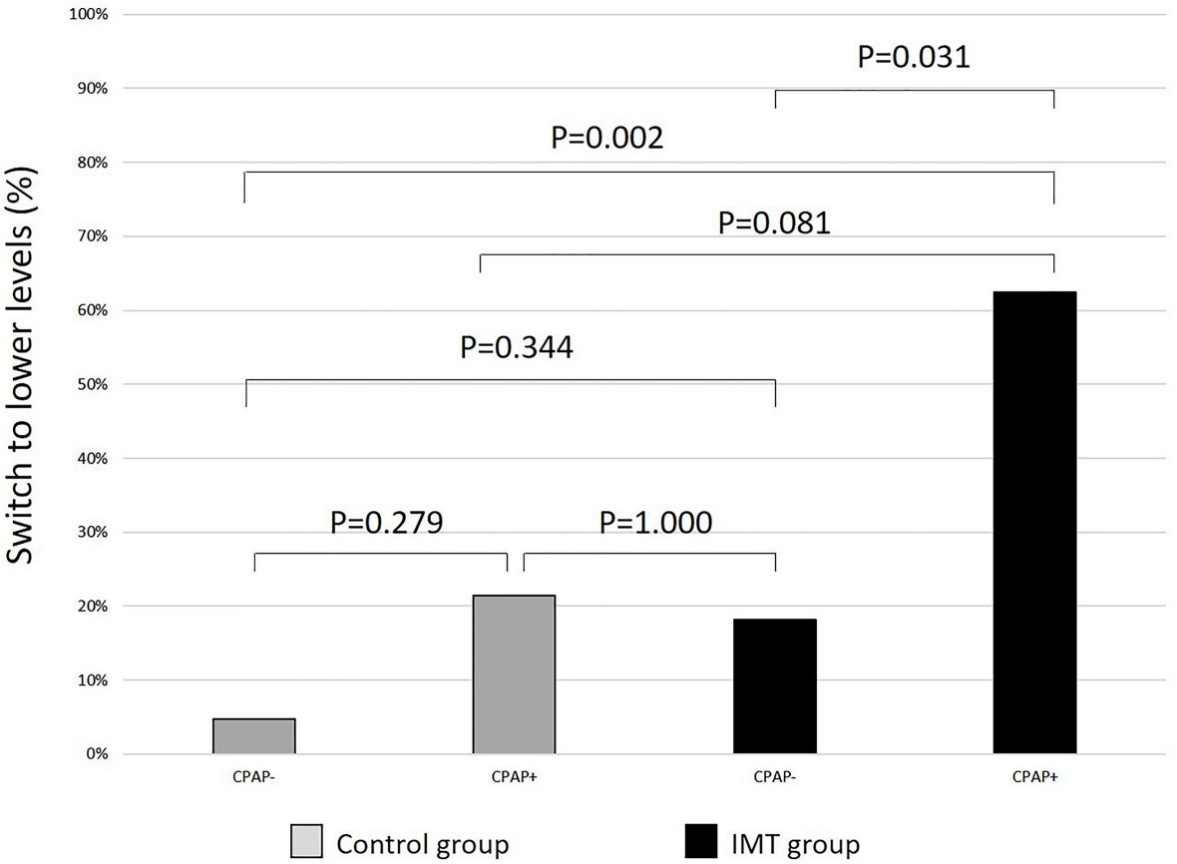
Percent of participants who progressed from the stage of severe obstructive sleep apnea (OSA) to lower levels in the subgroups that did not use continuous positive airway pressure (CPAP−) and that used CPAP (CPAP+) in the control group (gray bars) and the inspiratory muscle training (IMT) group (black bars). Differences tested by Friedman ANOVA complemented by Mann-Whitney test.

Data regarding the severity of OSA at baseline and at the end of the study are shown in Table 3. The frequency of severe OSA (events/hour >30) tended to decline in both subgroups of trained participants, but statistical significance was restricted to the ones who were undergoing CPAP treatment (50 *vs* 32%, P=0.358, and 87 *vs* 25%, P=0.041 for non-CPAP users and CPAP users, respectively). The percent of cases that decreased the levels of OSA severity was significantly higher in the subgroup of trained CPAP users compared with both the subgroup of non-trained/non-CPAP users (63 *vs* 5%, P=0.002) and the subgroup of trained/non-CPAP users (63 *vs* 18%, P=0.031) (Figure 2).

**Table 3:** Severity of OSA at baseline and at the end of the inspiratory muscle training program in the studied groups.

OSA severity, events/h	Control				Inspiratory muscle training			
	CPAP− (n=21)		CPAP+ (n=14)		CPAP− (n=22)		CPAP+ (n=8)	
	Baseline	Follow-up	Baseline	Follow-up	Baseline	Follow-up	Baseline	Follow-up
Mild (5-14.9)	8 (38)	7 (33)	2 (14)	5 (36)	1 (5)	4 (18)	0 (0)	2 (25)
Moderate (15-29.9)	8 (38)	10 (48)	2 (14)	0 (0)	10 (45)	11 (50)	1 (13)	4 (50)
Severe (>30)	5 (24)	4 (19)	10 (72)	9 (64)	11 (50)	7 (32)	7 (87)	2 (25)*

Data are reported as n (%). *P<0.05 *vs* baseline of the same subgroup. OSA: obstructive sleep apnea; CPAP(+/-): with or without continuous positive airway pressure.

## Discussion

We hypothesized that an IMT program could improve sleep patterns and reduce OSA severity. Significant variations in the 4 measured parameters throughout the study were found after intervention in both CPAP− or CPAP+ participants: PImax was increased, AHI was reduced, and improvements were seen in both ESS and PSQI.

Most studies that previously addressed the issue of IMT in OSA were performed with a small sample of 8 to 20 patients in each group (18–23), which warrants caution when considering negative numbers. In addition, the applied load and frequency of training were not uniform, nor was the duration of the IMT program, which ranged from 6 to 12 weeks. In this setting, the heterogeneity of the results cannot be seen as surprising. Of note, only one RCT focusing on the effect of IMT on blood pressure control in OSA had enrolled patients on CPAP therapy (23).

Overall, baseline data of the subgroups were comparable (Table 2). Not surprisingly, however, patients who were on CPAP at enrollment had a more severe form of OSA, in both the control and IMT groups. Variations in the four studied parameters throughout the present study after IMT were substantial. When analyzing PImax changes, there was significant muscle strength gain in the intervention subgroups, regardless of CPAP use. Measurements of PImax at baseline and at the end of study were carried out in three studies (18,21,22). Our findings were consistent with those studies, which reported significant muscle strength gain with IMT. In two of them, the strength gain was higher in the IMT group, similar to our findings (18,22).

A significant AHI reduction in our study was only found in the IMT subgroups, regardless of CPAP use. Three out of four previous RCT addressing IMT in OSA reported changes in AHI similar to our findings (19,20,22). The short duration of the IMT program (6 weeks) could have accounted for the negative findings in the Vranish and Beiley study (18).

When assessing aspects related to daily sleepiness, we identified a statistically significant reduction in ESS scores in CPAP users who were not trained, a finding that can be attributed to the CPAP therapy itself (11,26). In addition, statistically significant improvements in ESS scores were found in both IMT subgroups, regardless of CPAP use. This parameter was evaluated in 4 RCT in OSA patients with apparently conflicting results (19–22). Silva et al. (19) reported no changes along the study, but both groups were within the normal range at baseline making their findings difficult to interpret. Lin et al. (20) reported improvement in ESS scores restricted to the IMT group, but their control group was small, less than half of the intervention group. Finally, Souza et al. (21) and Nóbrega-Júnior et al. (22) reported improvements of the ESS along the study, but no difference between groups.

In the present study, the quality of sleep, as assessed by the Pittsburgh scale, significantly improved in participants who underwent IMT, again regardless of CPAP use. Data regarding the benefits of an IMT program on the quality of sleep are less conflicting. Consistent with our findings, every reviewed RCT reported significant improvement in PSQI score in participants undergoing IMT (18–22).

Specifically, IMT aims to improve the performance of the main and accessory respiratory muscles (19–22). The effects could occur independently of increases in muscle mass or strength via increased tone of pharyngeal muscles, which would reduce the tendency for upper airway collapse during sleep (11,13,14,19). In the present study, the starting point seems to have been an increase in respiratory muscle strength documented by an increase in PImax after IMT. This allowed a reduction in AHI and an improvement in sleep quality, resulting in positive effects on daytime sleepiness. It should be emphasized that all changes observed in the present study took place in a context where there were no changes in BMI and neck circumference in either the control or intervention groups.

One of the focuses of the present study was the impact of an IMT program on OSA severity. In this regard, the most impressive results took place in the subgroup of trained participants who were undergoing CPAP treatment (Table 3). The percent of cases that decreased the level of OSA severity in the trained participants on CPAP therapy were statistically higher than in the two subgroups not using CPAP, regardless of IMT, supporting the idea that the association of these two treatment strategies may be more beneficial.

As far as we know, this is the largest RCT to address this issue and the first to analyze the role of IMT as an adjuvant treatment for OSA in patients on CPAP. However, the study had some limitations. Data collection lasted longer than initially programmed. Like everyone else in the world, we were surprised by the onset of the COVID-19 pandemic, which led to the early completion of the study. The number of subjects who voluntarily dropped out in both segments of the study was not negligible (9 in the intervention group and 11 in the control one). This could be due to difficulty in complying with the protocol or returning to visits. Study dropout and stratification may have contributed to decreasing the power of the study to detect changes in some within- and between-group comparisons. Also, due to the nature of the study, blinding was only possible for the polysomnography technician, participants, and the researcher in charge of data analysis. Caution should be exercised regarding the generalizability of our findings due to the exclusion of patients with uncontrolled hypertension. Definitely, a more extensive study is necessary to substantiate our findings.

In conclusion, we found that a twelve-week program of inspiratory muscle training increased inspiratory muscle strength, substantially reduced AHI, and had a positive impact on sleep quality and daytime sleepiness. Findings occurred regardless of whether or not participants were using CPAP. Our findings reinforced the role of an inspiratory muscle training program as an adjunct resource in the treatment of OSA.
